# Age as a Risk Factor in the Occurrence of Complications during or after Bronchoscopic Lung Biopsy

**DOI:** 10.3390/geriatrics7020034

**Published:** 2022-03-21

**Authors:** Irina Pocienė, Rasa Gauronskaitė, Domantas Galkauskas, Antanas Mainelis, Vygantas Gruslys, Edvardas Danila

**Affiliations:** 1 Centre of Pulmonology and Allergology, Vilnius University Hospital Santaros Klinikos, 08661 Vilnius, Lithuania; rasa.gauronskaite@santa.lt (R.G.); domantas.galkauskas@santa.lt (D.G.); vygantas.gruslys@santa.lt (V.G.); edvardas.danila@santa.lt (E.D.); 2 Clinic of Chest Diseases, Immunology, and Allergology, Faculty of Medicine, Vilnius University, 03101 Vilnius, Lithuania; 3 Faculty of Mathematics and Informatics, Vilnius University, 03225 Vilnius, Lithuania; amgpastas@gmail.com

**Keywords:** bronchoscopy, lung biopsy, pulmonary complications, elderly patients

## Abstract

Introduction: Bronchoscopic lung biopsy (BLB) is a widely used procedure. As the world’s population is ageing, more BLBs are performed for older people with comorbidities. The aim of the study was to investigate if an older age is a risk factor for BLB related complications. Materials and Methods: A prospective study at the Centre of Pulmonology and Allergology of Vilnius University Hospital Santaros klinikos was conducted. Seven hundred and eighty-six patients (male 60.6%), mean age 57 ± 16, who underwent BLB, were included. The complications that occurred due to BLB were evaluated. Bleeding and pneumothorax were classified into I° or II° grades depending on their severity. Potential determinants, which may increase the risk of complications, emphasizing on age, were analyzed. Results: Fifty-seven (7.2%) BLB-related complications occurred. There were 27 (3.4%) pneumothoraxes, and 19 (70%) of them required thoracic drainage. Thirty (3.8%) bleeding complications occurred, and four (16%) of them were severe. Higher rates of bleeding were found in the age group ≥65 years, *p* = 0.001. The risk of bleeding in older patients was 3.2 times higher (95% CI 1.51–6.87). Conclusions: Older age is related to a higher incidence of mild bleeding during BLB. However, the risk of life-threatening complications is low despite the age, and older age should not be considered as a contraindication for the procedure if needed.

## 1. Introduction

Bronchoscopic lung biopsy (BLB) is a procedure that has been used to diagnose a wide spectrum of lung disorders for more than half of a century [1]. Although high resolution computed tomography, pulmonary function tests and bronchoalveolar lavage are useful for diagnosing various lung diseases, BLB remains an important diagnostic tool in the majority of cases, especially lung cancer and interstitial lung diseases. New alternative diagnostic techniques, such as endobronchial ultrasound-guided transbronchial needle aspiration (EBUS-TBNA), CT-guided transthoracic needle biopsy (PCNB) and bronchoscopic lung cryobiopsy (BLC) have a specific area of use and yet are not universal. For this reason, BLB is still relevant and widely used in daily practice [2].

There are already studies evaluating the risks associated with bronchoscopic lung biopsies, focusing on the overall rate of complications, patients with lung diseases (e.g., lung cancer, interstitial lung diseases), immunocompromised patients, various laboratory findings, etc. [3,4,5,6]. As the world’s population is aging, more diagnostic BLBs are performed for older people who have comorbidities and are taking several medications [7]. However, to this day, there is a lack of studies regarding BLB using a flexible bronchoscope complications rate in older patients, especially in those with comorbidities [8].

The aim of the study was to investigate if an older age is a risk factor in the occurrence of BLB related complications, focusing on bleeding, pneumothorax, cardiovascular events, severe infection and death. The study also aimed to assess other possible risk factors of BLB complications, with an emphasis on concomitant diseases.

## 2. Materials and Methods

From 2009 to 2016, a prospective study at the Centre of Pulmonology and Allergology of Vilnius University Hospital Santaros klinikos was conducted. Seven hundred and eighty-six consecutive patients who underwent BLB using a flexible bronchoscope were included in the study. Complications that occurred during or within 24 h after BLB were evaluated. Potential determinants of BLB complications, such as age, comorbidities, anticoagulant therapy usage, platelet count, location of a lesion and the number of specimens taken, were also analyzed.

In our study population, the main indications for BLB were peripheral lung nodule(s), opacity(ies) and diffuse parenchymal changes (reticular, ground-glass opacities, etc.) in the lungs, usually with suspicion of lung cancer or interstitial lung disease. Following the guidelines [9], severe hypoxemia (need for non-invasive or mechanical ventilation), unstable severe obstructive airway disease, severe pulmonary hypertension, hemodynamic instability and myocardial ischemia were contraindications for the procedure. In our center, older age and other comorbidities (not listed above) per se were not considered as contraindications for BLB.

**Figure 1:**
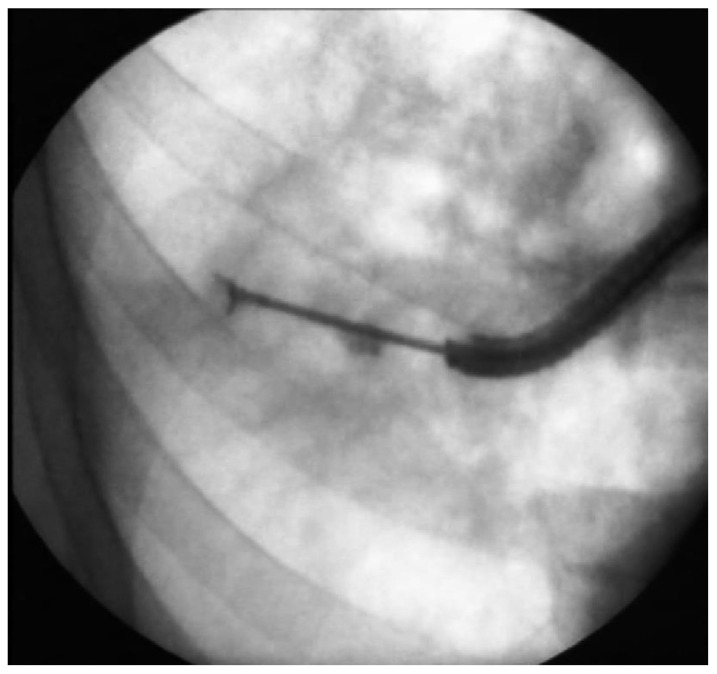
Fluoroscopy-guided bronchoscopic lung biopsy.

Fiberoptic bronchoscopy and BLB were performed as described elsewhere [10,11]. Patients were premedicated with atropine, and lidocaine was delivered topically via an atomizer. The bronchoscope was inserted transnasally (in most cases) or orally. After the inspection of the tracheobronchial tree, a bronchoscope was inserted to subsegmental or smaller bronchus until the wedging position. Under fluoroscopic control, biopsy forceps were pushed forward until mass, nodule or a peripheral position in the case of diffuse parenchymal lung changes. The position of biopsy forceps was controlled by two directions of chest fluoroscopy. Afterward, the forceps were withdrawn about 2–3 cm, then opened and pushed forward. Usually, this maneuver was repeated once or twice; then, the forceps were closed and withdrawn (see Figure 1 [12]. In the case of diffuse lung changes, the BLB was performed after a patient inhaled. In the case of a solitary nodule or an infiltrate, the BLB was performed independent of the breathing phase.

All patients who underwent BLB were further supervised as required in our local protocol for up to 24 h. Vital signs (respiratory rate, oxygen saturation, blood pressure, pulse rate, body temperature, ECG) were monitored during the first 2 h and prolonged if needed. A chest X-ray was performed the next morning for all the patients. If there was a suspicion of pneumothorax (dyspnea, chest pain), an X-ray was performed immediately [8].

Bleeding severity was classified as follows: I° grade—bleeding, which required intrabronchial saline or/and adrenaline injection, temporal bleeding segment occlusion with the bronchoscope or discontinuation of the procedure (smaller number of specimens were taken) [13]; II° grade—bleeding, required obturation of the bronchi using a balloon catheter or polyurethane foam, blood transfusion or patients’ administration to the intensive care unit (intubation and mechanical ventilation). Pneumothorax severity was divided into two grades: I° grade—no interventions needed (spontaneous resolution); II° grade—required thoracic drainage [14].

Descriptive and group comparison statistical analysis was performed using SPSS version 20 (SPSS Inc., Chicago, IL, USA). The mean, standard deviation, minimum and maximum values of a quantitative variable age were presented. Categorical variables were presented as the absolute amount and the percentage. In order to test the hypothesis for a between-group comparison of the categorical variables, Pearson’s Chi-Square or Fisher‘s exact tests were used as appropriate. Odds ratios and 95% confidence intervals were calculated for the estimating relations of groups and other binary variables where it was possible, and the statistical significance was found. For comparison of complications of biopsy between side diseases, *p*-values adjusted for age and gender were calculated using rare event logistic regression from package ‘Zelig’ of statistical program R (v3.5.1). A *p*-value less than 0.05 was considered significant.

This research was conducted in accordance with all relevant guidelines and procedures. The study was approved by the Vilnius Regional Biomedical Ethics Committee (No. 158200-13-652-210).

## 3. Study Population

Seven hundred and eighty-six patients were included in the study, 476 men (60.6%) and 310 women (39.4%). Statistically, no significant differences between men and women were found. The main indication for BLB was pulmonary nodule or mass (47.5% of cases). The second most common indication was diffuse lung changes (33% of cases). In 373 (47%) cases, the biopsy was taken from the upper lobes of the lungs. On average, 10 ± 3 specimens were taken during the procedure.

The mean age of the study population was 57 ± 16 years, with the youngest person of age 18 and the oldest of age 92. There were 94 (12%) patients taking drugs with an effect on the coagulation system. The mean patients’ platelet count before the procedure was 270 ± 101 × 10^9^/L. All patients were divided into two age groups (<65 and ≥65 years) and nine groups according to comorbid diseases for better assessment of factors possibly increasing the risk of BLB complications. Age 65 was set as the threshold of old age since at this period of life, the rates for sickness and death begin to show a marked increase [15]. Demographic data are summarized in Table 1.

## 4. Results

**Figure 2:**
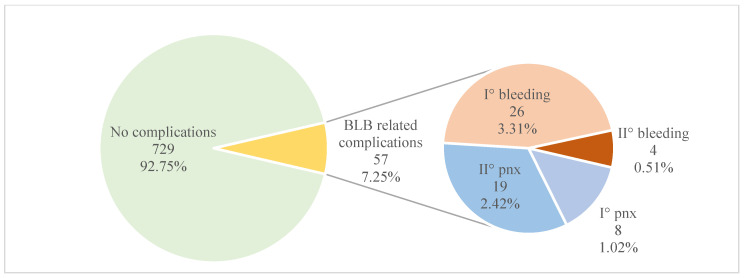
Complications of the bronchoscopic lung biopsy procedure. pnx—pneumothorax.

In total, 57 (7.2%) complications of BLB occurred. There were 27 (3.4%) pneumothoraxes and 30 (3.8%) bleedings. Most of the pneumothoraxes (19; 70%) required chest drainage, and most bleedings (26; 86%) were classified as mild (grade I°). Summarized data are shown in Figure 2 and Table 2. One of the patients had both complications: I° bleeding and II° pneumothorax. No deaths or cardiac events occurred during or after the procedure. Two patients had severe infections after the procedure, where intravenous antibiotics were needed (final diagnoses—lung cancer, complicated by pneumonia).

Higher rates of bleeding were found in the age group ≥ 65 years compared with the age group < 65 years old, *p* = 0.001. The odds of bleeding were 3.2 times higher in older patients (95% CI 1.51–6.87). On the contrary, no relationship was found between older age and pneumothorax due to BLB.

Patients with cardiovascular diseases (Group I) had a higher risk of bleeding during BLB compared with other groups of comorbidities (*p* = 0.019) when applied to all the study population. As most of the patients with cardiovascular diseases were older than 65 years, when adjusted to age (≥65 or <65), no statistical significance between higher bleeding rates and the presence of cardiovascular diseases was found. The conclusion was made that higher bleeding rates were related to age and not to the presence of cardiovascular diseases.

Unexpectedly, no relationship was found between antiplatelet or anticoagulant usage or low platelet count and bleeding. Radiological changes, even fibrosis and emphysema of the lungs or location of those changes did not increase the risk of BLB complications. Moreover, the number of specimens taken during the procedure was not related to the increased rate of complications—taking even up to 13 specimens on average did not increase the risk of bleeding, pneumothorax, or other serious complications.

## 5. Discussion

Our study demonstrates BLB complication rates and factors associated with most common procedure-related complications, specifically pneumothorax and pulmonary hemorrhage. This study shows that older patients (≥65 years old) have an increased risk of BLB related hemoptysis. However, despite the age, most of the complications usually are mild and BLB, in general, is a safe procedure with a low risk of life-threatening complications and outcomes.

Rates of BLB related complications highly varies among different studies. Estimates of risk of pneumothorax usually range from 0% to 5% [16,17,18]. Serious BLB related hemoptysis occurs approximately in 0.6–1.3% of cases [14,17,18]. Some case studies of immunocompromised patients demonstrate the incidence of bleeding as high as 26% [3].

In our study, the overall rate of BLB related pneumothorax was 3.4%, and procedure-related bleeding occurred in 3.8% of cases. Compared with other studies, our rates of BLB related pneumothorax, including the need for chest tube placement, were similar. In contrary to pneumothorax, the comparison of bleeding rates due to BLB is more complicated.

Definition of hemorrhage during BLB is less standardized, as blood is usually diluted with other fluids, e.g., lidocaine, saline, sometimes even bronchial secretions. Some authors measure bleeding volume during the BLB procedure in milliliters [3]. Others, including our study, judge bleeding as clinically significant by the need for clinical intervention [14]. We considered the latter type of classification was better as the volume of clinically significant bleeding also varies among different studies. Our data showed that bleeding was rare, and it was easily managed without serious adverse outcomes in most cases.

We additionally searched for risk factors associated with increased risk of complications due to BLB, with an emphasis on age, as older patients usually have more comorbidities and use a variety of medications, which may increase the occurrence of procedure-related complications. It was found that the risk of BLB related bleeding was 3.2 times higher in older patients. The study in the U.S., where databases from 2000 to 2009 were analyzed, also found that age was significantly associated with BLB related hemoptysis [18]. However, other studies did not find any significant age and bleeding relationship [16,19,20].

We made further analysis trying to find out possible reasons why older age was related to higher bleeding rates in our study. In the elderly, higher bleeding rates during invasive procedures may be associated with medications that have an effect on the coagulation system. Usually, people older than 65 years are at higher risk of thromboembolic disorders, so many of them use anticoagulants and antiplatelet drugs to prevent or treat thromboembolic events [4]. However, our study showed no relationship between bleeding rates during BLB and anticoagulant and antiplatelet drug usage when they were temporarily discontinued before the procedure. Anticoagulants were discontinued as it is recommended in the summary of product characteristics (SPC) of the particular drug. Our decision to temporarily withhold antiplatelet drugs before the procedure was quite strict as, to this day, there is a lack of studies addressing the performance of BLB in patients taking these medications. One comparative study [14] showed that aspirin usage did not increase the risk of bleeding during BLB. However, based on our observations and experience, we consider it is more reasonable to withhold antiplatelet drugs, if possible.

Thrombocytopenia is another well-known risk factor for hemorrhage during invasive procedures [4]. Despite the lack of data, it is considered safe to perform BLB when the platelet count is greater than 50.000/mm^3^ [21]. Following this guideline, all BLB were performed with platelet counts higher than 50.000/mm^3^ in our department. Platelet count was similar in different age groups, and it was not related to an increased risk of bleeding. Thus, this factor did not explain higher bleeding rates in the elderly either.

Uremia may also increase the incidence of bleeding during invasive procedures as it affects platelet function [22]. Moreover, renal function declines because of the natural physiological course of aging [23], so the risk of bleeding further increases. However, laboratory findings and their possible relation with BLB complications were not our primary aim of the study. We did not check serum creatinine or BUN levels before the procedure unless it was known that the patient had chronic kidney disease. It remains unclear if a worse renal function had any influence on higher rates of hemorrhage during BLB and older age in our study. Despite that, we did not find any relationship between chronic kidney disease and higher bleeding rates during the BLB procedure.

Perception of the risk of complications due to BLB when there are certain comorbidities highly varies between pulmonologists [24]. Some studies suggest that a higher incidence of hemoptysis during invasive procedures may possibly be related to comorbidities [25] the patient has. That is the reason we distinguished nine different groups of medical conditions. Patients with cardiovascular diseases had a higher risk of bleeding during BLB, but no significant relation was found when adjusted to age. Thus, we assumed that the risk of bleeding was higher in this group because of age and not because of cardiovascular diseases. In our study, other comorbidities also had no impact on the bleeding rate during BLB. Comorbidities also had no influence on the increased risk of pneumothorax either. The decision was made that age was independently related to the increased risk of bleeding during BLB in our study.

There is a lack of data on the optimal number of specimens taken when performing BLB and if it may increase the risk of complications during the procedure. Taking at least 6 samples for the peripheral mass or infiltrate and up to 10 samples for diffuse changes is recommended [20,26]. Some studies show that a higher number of specimens are needed for maximum diagnostic yield, especially for peripheral lung changes [27]. We found that a higher number of specimens taken during the BLB procedure did not significantly increase the risk of complications. Our study suggests that it is safe to take even up to thirteen specimens during BLB if it is necessary, but further investigation is needed to assess if it increased the diagnostic yield of the procedure.

Death due to BLB is uncommon [28]. However, it is thought that death cases may be under-reported [19]. In our study, there were no deaths due to BLB, and it strongly suggests that BLB is a safe procedure.

## 6. Strengths and Limitations

Our study has its strengths and limitations. The study was conducted prospectively. This is one of its major strengths, as BLB related complications were defined before the study began. Moreover, all bronchoscopic lung biopsies were performed by the same team of experienced specialists using the same technique of the procedure. The large study population is another advantage of our study. Other researches, which investigated BLB related complications, are either smaller in size or retrospective, usually based on surveys or databases [18,24]. We also only had a few absolute contraindications for the BLB procedure. Our patients’ age range was wide, from as young as 18 years to as old as 92 years, with various comorbidities and medications usage, which means that our study population reflects the cases doctors face in their daily practice.

A potential limitation of our study is that all BLB procedures were performed by experienced interventional pulmonologists in a well-equipped tertiary center. Therefore, the results may not be applicable to smaller hospitals. Another possible study limitation is that lung function tests were not routinely performed before the procedure. Studies show that forced expiratory volume in 1 s (FEV_1_) reduction may reflect a prevalence of emphysema and, therefore, may increase the risk of pneumothorax during BLB [29,30]. As FEV_1_ was not known in the majority of our patients, we did not evaluate the relationship between lung function and BLB related pneumothorax. Another disadvantage of the study is that in most of the cases, we did not check serum creatinine or BUN levels, which may have a relation with BLB related hemorrhage.

## 7. Conclusions

Older patients have an increased risk of bronchoscopic lung biopsy-related bleeding. However, in most cases, the bleeding is mild and does not require additional clinical interventions. Therefore, in general, bronchoscopic lung biopsy is a safe procedure with a low risk of life-threatening complications. Neither older age nor concomitant diseases are contraindications for the procedure if clinically necessary.

## Figures and Tables

**Table 1:** Study population.

	<65 Years Old (N = 503; 63.9%)	≥65 Years Old (N = 283; 36.1%)
Comorbidities (number (%))		
Group I (cardiovascular diseases)	145 (28.8)	190 (67.1)
Group II (BA, COPD, bronchiectasis) *	44 (8.7)	60 (21.2)
Group III (chronic respiratory failure) **	15 (3)	15 (5.3)
Group IV (stroke)	5 (1)	13 (4.6)
Group V (other neurological diseases)	10 (2)	18 (6.4)
Group VI (endocrine disorders)	23 (4.6)	29 (10.2)
Group VII (renal diseases)	5 (1)	9 (3.2)
Group VIII (non-pulmonary cancer)	23 (4.6)	36 (12.7)
Group IX (hematological diseases) ***	26 (5.2)	10 (3.5)
Group X (no comorbidities)	289 (57.5)	55 (19.4)
Multimorbidity (≥2 comorbid diseases)	62 (12.3)	104 (36.7)
Medications (number (%))		
LMWH ****	22 (2.8)	23 (2.9)
Warfarin	17 (2.2)	6 (0.8)
Aspirin	17 (2.2)	9 (1.1)
Platelet count (mean ± standart deviation, × 10^9^/L		
	267 ± 102	219 ± 45

* BA—bronchial asthma, COPD—chronic obstructive pulmonary disease. ** Need for supplemental O_2._ *** Hodgkin’s lymphoma, non—Hodgkin’s lymphoma, leukemia, thrombocytopenia. **** LMWH—low molecular weight heparin.

**Table 2:** Patients’ age and complications rate.

		Bleeding N (%)	*p*	Pneumothorax N (%)	*p*
Age	<65 (503; 64%)	11 (36.7)	0.001	17 (63)	0.909
	≥65 (283; 36%)	19 (63.3)		10 (37)	

## Data Availability

Data are available from the corresponding author Irina Pocienė (irina.liustrickyte@santa.lt) on reasonable request.
